# Effect of Traditional Cooking and Sous Vide Heat Treatment, Cold Storage Time and Muscle on Physicochemical and Sensory Properties of Beef Meat

**DOI:** 10.3390/molecules27217307

**Published:** 2022-10-27

**Authors:** Marian Gil, Mariusz Rudy, Renata Stanisławczyk, Paulina Duma-Kocan

**Affiliations:** Department of Agricultural Processing and Commodity Science, Institute of Food and Nutrition Technology, College of Natural Sciences, University of Rzeszow, St. Zelwerowicza 4, 35-601 Rzeszow, Poland

**Keywords:** beef meat, sous vide, cooking, cold storage, sensory properties

## Abstract

Consumers are avoiding the consumption of highly processed foods, aware of the negative effects of the additives or high temperatures used on the biological value of the food. This causes an interest in ways of minimal processing or low-temperature cooking procedures. However, to achieve the desired organoleptic quality, it is necessary to know the relationship between the parameters of the treatments and the type of raw material. The purpose of this study was to investigate the complex effects of traditional cooking and sous vide heat treatment, cold storage time and muscle on the physicochemical and sensory properties of beef. The study material consisted of samples of musculus longissimus thoracis and musculus semitendinosus obtained from beef half-carcasses. The muscles were subjected to traditional cooking in water at 95 °C until the temperature inside the piece reached 65 °C and sous vide treatment at 65 °C for 2 h. The study was performed after 2 and 21 days of cold storage. Instrumental evaluation of texture parameters, color and sensory evaluation of meat was carried out. Meat stored for 21 days was characterized by more favorable TPA test (Texture Profile Analysis) results compared to meat evaluated 48 h post mortem. The study also showed positive effects of sous vide heat treatment on texture parameters and sensory properties (especially on tenderness and palatability), as well as differences in the formation of quality traits between muscles. Given the trends associated with energy-saving technologies, it is desirable to seek the optimal combination of temperature and time of fixation treatments at an acceptable level of quality. The use of low-temperature cooking for as little as 2 h, yields positive results in sensory evaluation of juiciness, tenderness, or palatability.

## 1. Introduction

Beef is most often consumed after heat processing. Consumers are increasingly avoiding highly processed meat products, especially those heated to high temperatures during frying, roasting, or grilling. During these thermal treatments, many Maillard reaction products are formed [1], which have a positive effect on the palatability of meat products but have harmful (or at least controversial) effects on human health [2,3].

Color is the most important of the quality parameters affecting the marketability of fresh beef, as at the point of sale, consumers often associate the bright cherry-red color of fresh beef and the purple-red color of vacuum-packed beef as indicators of wholesomeness [4]. The internal color of cooked beef is used as an indicator of frying level and safety when consumed [5].

Thermal preparation of meat is very important to increase the palatability, digestibility and safety of the product [6,7]. The cooking method is considered the last element in the process of shaping the final quality of beef [8]. The available literature indicates that the proper selection of the final cooking method is crucial to the tenderness, flavor and juiciness of beef and should be selected individually [9,10,11].

The palatability of beef is generally determined by three quality attributes that are experienced during meat consumption—flavor, tenderness and juiciness [12,13,14]. Attributes vary and depend on a number of factors: animal production—breed, genetics, diet, animal age, susceptibility to stress, etc.; carcass processing—degree of quality, marbling, maturation, electrostimulation, cooling methods, carcass hanging method, product refinement, etc.; and the final preparation and cooking procedures applied to the product [8,13,14,15].

Meat tenderness is a major determinant of consumer satisfaction and the likelihood of purchase. A varying supply of tender meat negatively affects consumer demand and acceptance [15]. Tenderness is determined by a complex interaction of live and post-slaughter factors. These factors range from practices used throughout the animal production chain, i.e., genetics, animal breeding, animal nutrition, housing, transportation, stunning and bleeding, to meat storage methods and final product cooking procedures [16]. Maturation of meat used in the meat industry to improve meat tenderness raises the energy consumption needed for cold storage and thus production costs [17]. Improving beef quality and its reproducibility is the goal of studies by many research groups [8,11,18].

Cooking coagulates and denatures meat proteins, improves meat palatability, destroys a significant number of microorganisms, improves the shelf life of meat products, deactivates native proteolytic enzymes, prevents unpleasant flavors and modifies the texture or tenderness of meat and meat products [19].

New technological developments have led to innovative thermal and non-thermal treatments to improve meat tenderness [15,20].

The composition of meat tissues has changed over the past few decades toward more lean elements. Traditional thermal processing methods are no longer the best option for preparing meat products, as they often do not provide sufficient juiciness and flavor. In contrast, the sous vide method makes it possible to obtain juicy, tender and flavorful products from almost any carcass cut [21]. It should be noted that in order to improve the tenderness and juiciness of various meats [22], especially beef [3,15,23,24], the use of the sous vide method is recommended [8].

Sous vide (SV) cooking is achieved by heating meat at a lower temperature for a longer period of time, which can induce ideal changes in meat characteristics and preserve the more natural condition, moisture content, nutritional value and flavor of various meats, including beef [25], lamb [26] and pork [27,28,29]. SV cooking, compared to conventional cooking, allows a higher activity of endogenous enzymes (cathepsin and calpain), which are responsible for meat tenderness by increasing the solubility of collagen [29,30].

SV cooking is an example of a long-term low-temperature heating method. In particular, the SV cooking process is more economical in terms of heat, and the technology allows precise temperature control over any range used for cooking purposes [31]. SV cooking differs from traditional cooking methods in that the raw food is vacuum-packed in thermostable food-grade plastic bags, and the food is cooked using precisely controlled heat. Vacuum sealing has several advantages: it allows efficient heat transfer from water (or steam) to the food; it increases food shelf life eliminating the risk of recontamination during storage; and it prevents oxidation of unpleasant aftertastes and prevents evaporation loss of flavor volatiles and moisture during cooking [21]. Sous vide protects meat products from surface dehydration compared to traditional cooking, or heating in dry air [32,33]. In addition, sous vide reduces color changes and the formation of heterocyclic aromatic amines in meat products compared to pan-frying [34].

Sous vide meat not only has better sensory properties but also better-preserved nutritional quality. Sous-vide cooking is increasingly being used to prepare beef because it helps reduce the difference in the tenderness of products obtained from cattle of different ages and sexes, as well as different meat maturation times [22,25,35].

Cooking in vacuum-sealed bags ensures microbiological safety [36], and also prevents lipid oxidation [26,37,38], which is a major cause of product deterioration [39]. In recent decades, sous vide cooking has become more popular and widely used in restaurants and culinary schools. Equipment costs initially limited access for home users, but the advent of inexpensive clamp-on circulators has increased the availability of this method for home users testing the technology [40].

A lot of work has also been devoted to studying the effect of low-temperature long-term heating on meat quality. There is no doubt that such a method of treatment is beneficial for the organoleptic quality of meat, but taking into account the time of treatment and the amount of energy consumed for this purpose, this method also increases the price of the product. It seems reasonable to search for the optimal combination of temperature and treatment time at an acceptable level of quality.

The aim of the present study was to investigate the complex effects of traditional cooking and sous-vide heat treatment, cold storage time and muscle on the physicochemical and sensory properties of beef.

## 2. Results

The texture parameters and hydration properties of raw beef depending on the type of muscle and cold storage time are shown in Table 1. These data show that muscle had an impact on the variation of meat texture parameters such as bumpiness, cohesiveness, resilience and chewiness. While cold storage time affected adhesiveness, thermal leakage and forced leakage. In turn, muscle, cold storage time, and the interaction effect between the two factors had a statistically significant effect on cutting force.

Cycle 1 hardness of raw semitendinosus (MS) muscle took higher values both after 2 days and after 21 days of storage compared to m. longissimus thoracis (MLT). Muscles stored for 21 days were characterized by lower values of this parameter. No statistically significant differences were found for hardness 1 and hardness 2. The adhesiveness of MS was lower compared to that of MLT, and it increased in both muscles after 21 days of storage. In contrast, the bumpiness in MS took higher values compared to MLT, and storage time did not cause significant differences. The cohesiveness of MS was statistically significantly higher compared to MLT at both test dates, and its value increased after 21 days of storage, but only for MS. The resilience of MS was higher compared to MLT (but the differences were statistically insignificant). The resilience of MLT decreased (*p* < 0.05) during the storage. Gumminess and chewiness values were higher in MS, and the differences were statistically significant for chewiness.

The cutting force of raw meat of MS was 123.61 N and increased (*p* < 0.05) after 21 days of storage to 157.94 N. MLT had a statistically significantly lower cutting force value compared to MS at both test dates. Forced leakage describing the ability of meat to hold water in both muscles was similar, ranging from 6.07 to 6.89 cm^2^ in meat 2 days post mortem. Muscle maturation resulted in improved water-holding capacity, with thermal leakage decreasing (*p* < 0.05) from about 32% to about 24%, and forced leakage decreasing to 4.03–4.18 cm^2^ after 21 days of cold storage.

The brightness of the color of the raw MS took higher values compared to MLT on both test dates (Table 2). The a*, b* parameters of the MS also took higher values. Storage caused an increase in the values of these parameters (except for the MS) with a statistically significant increase in the MLT. The calculated absolute color difference due to storage time, as well as the type of muscle, indicates a significant color difference in the evaluated raw material (ΔE > 5).

The results concerning texture parameters of beef cooked by traditional and sous vide methods depending on the type of muscle and cold storage time are presented in Table 3. These data show that the level of meat texture parameters such as cycle 1 hardness, bumpiness and gumminess were influenced by the interaction between the type of muscle, its storage time before treatment and the type of thermal treatment used. Cohesiveness, resilience and cutting force depended on the interaction of treatment method and storage time. In the case of resilience, the interaction of the aforementioned factors with the type of muscle also played a role.

The hardness 1 and hardness 2 values of sous vide cooked meat were almost twice as high (*p* < 0.05) in MS meat compared to MLT after 21 days of cold storage. The differences in the values of these characteristics between the other groups were statistically insignificant. The values of the bumpiness parameter in both traditionally cooked muscles were similar on both test dates. In MS muscle cooked by the sous vide method, a reduction (*p* < 0.05) in the bumpiness value was noted, but only after 21 days of cold storage. Sous vide cooking of muscle stored for 21 days resulted in a significant decrease (*p* < 0.05) in cohesiveness values compared to muscle after 2 days of storage. The resilience of both traditionally cooked muscles was similar. The use of the sous vide method showed slightly higher values for this parameter in MS on both test terms compared to MLT. There was also a reduction in the resilience values of both sous vide cooked muscles after storage for 21 days (however, the differences were statistically significant only for the MLT muscle). The gumminess of MS cooked after 2 days of cold storage was higher than in MLT, but after storage for 21 days the value of this parameter was similar in both muscles. A decrease in gumminess was noted in MLT stored for 21 days cooked by the sous vide method. However, the differences were statistically insignificant for this trait. Higher values (*p* < 0.05) of the cutting force of cooked meat were recorded for MS compared to MLT after both 2 days and 21 days of cold storage. Similar relationships also occurred in those muscles cooked by the sous vide method. The cutting force values required to cut a sample of meat cooked by the sous vide method were lower than those of meat cooked by the conventional method.

The results for the color parameters of traditionally and sous vide cooked beef depending on the type of muscle and cold storage time are shown in Table 4. MS cooked traditionally had higher L* brightness values compared to MLT, and for both muscles, there was a brightening of the color of the cooked meat after 21 days of storage (however, the differences were not statistically significant in each case).

The brightness parameter L* in sous vide cooked muscles took higher values than in conventionally cooked meat, but statistically significant differences in the values of the L* parameter between the two test terms were only found for MS muscle. The values of the a* parameter, which determines the level of red, were similar between the two test terms in traditionally cooked muscles. Muscles cooked sous vide showed twice as high (*p* < 0.05) values of the a* parameter after 2 days of storage as those of traditionally cooked muscles, while sous vide cooked muscles showed a slight decrease in values of this parameter after 21 days. The variation of this parameter among the groups is due to the existence of an interaction between the type of muscle and the heat treatment used and the interaction between the type of treatment and storage time. The values of the parameter b*, which expresses the level of yellow in both muscles, are differentiated by the interaction effect of storage time and type of muscle and storage time and heat treatment applied.

The value of the absolute color difference calculated for traditionally cooked muscle due to storage time indicates that there is a clear color difference between meat stored for 2 and 21 days. Such a color difference was also observed in sous vide meat, and in the case of MS muscle, the level of this parameter even indicates that the observer perceives two different colors depending on storage time. The absolute color difference calculated due to the type of heat treatment used indicates clearly perceptible to the observer different colors between MLT and MS stored at different times. The existence of observable color differences is also indicated by the levels of the absolute color difference calculated between the muscles tested.

The results for the sensory evaluation of beef meat according to the type of muscle, cold storage time and type of heat treatment are shown in Table 5. Scores for the intensity as well as the desirability of meat odor were slightly higher for muscles stored for 21 days compared to those cold stored for 2 days, regardless of the type of heat treatment.

Juiciness in traditionally cooked meat was rated slightly more favorably for MLT at both test dates. Sous vide cooking influenced higher juiciness evaluation for meat in both test terms and both muscles compared to traditional cooking. The tenderness of the MS muscle was generally rated more favorably compared to the MLT. Also, tenderness in meat stored for 21 days for both muscles was rated higher for both heat treatments. For both muscles, longer storage time affected the intensity and desirability scores of palatability slightly more favorably, especially for sous vide heat-treated meat. However, a statistically significant difference was noted only for the tenderness of sous vide-treated MLT muscle and cold storage of 2 and 21 days. The sous vide heat-treated meat received higher sensory evaluation scores compared to conventionally cooked meat.

## 3. Discussion

Both the type and time of thermal processing and the level of temperature, in addition to influencing the amount of culinary meat weight loss, affect organoleptic properties. Botinestean et al. (2016) [25] found lower cutting force values for beef cooked sous vide at 60 °C compared to beef heated at 70 °C. A study by Modzelewska-Kapitula et al. (2019) [3], showed higher cutting force values in meat cooked by steaming compared to sous vide cooked meat. Lorenzo et al. (2015) [41] indicated a relationship between cutting force values and thermal losses—samples with high thermal loss also had the highest cutting force values. The results of Naqvi et al. (2021) indicate a significant effect of temperature increase to 75 °C on reducing the cutting force values. The cited authors showed a positive effect of maturation on the reduction of cutting force values of biceps femoris and semitendinosus muscles. Cutting force values were lower in muscle matured for 13 days compared to 0 days in both muscles [15]. These results coincide with the works of Wyrwisz et al. [42] and Wu et al. [43]. These studies showed that the maturation process significantly increased the tenderness of beef meat as a result of proteolysis of myofibrillar proteins of longissimus dorsi and semimembranosus muscles. The effect of maturation can be explained by myofibrillar fragmentation. A process of meat softening that results from the activity of endogenous muscle enzymes is observed during maturation. In addition, as shown by Onopiuk et al. (2022), meat tenderness is significantly related to chemical composition. Maturation at cold storage temperatures (2 ± 1 °C) resulted in a decrease in meat tenderness values during storage. No significant change was found in meat hardness for m. semimembranosus even at day 5 post mortem. A significant reduction in cutting force was observed on days 10 and 15 [44]. Maturation of meat and proper selection of heat treatment parameters is particularly important for meat from older and lower-quality animals. A study by Naqvi et al. (2021) showed that meat from older cattle required higher temperatures and prolonged heat treatment to achieve similar tenderness to that observed in muscle from younger animals. Clear differences in cutting force values between younger and older animals were observed at 55 °C and 65 °C, but the difference decreased significantly at 75 °C in both biceps femoris and semitendinosus [15]. Similarly, the tenderness of meat evaluated sensorially in the present study (Table 5) was rated higher in meat aged 21 days compared to meat evaluated 2 days post mortem. Also, the type of processing differentiated the scores of this trait and the tenderness of meat cooked sous vide was rated higher. Research by Botinestean (2021) indicates that a sous vide cooking time of 120 min was sufficient to impart the desired texture characteristics to older consumers, regardless of papain concentration [45].

A temperature of about 60 °C appears to be a good choice for preparing beef sous vide. A study by Botinestean et al. (2016) showed that conducting sous vide at 60 °C reduces thermal loss, cutting force, hardness and chewiness of beef and improves appearance compared to a process conducted at 70 °C [25]. Beef subjected to sous vide at 60 °C also has lower cutting force values than beef heated at 50 °C [38].

Ensuring meat with consistent quality characteristics is particularly important due to recent trends in meat consumption, where price will not be the most important purchasing factor, and high and reproducible quality (nutritional, sensory and hygienic values) will be most important [12,46,47]. A tenderness that meets consumer expectations is a key parameter of organoleptic quality. O’Quinn et al. (2018) indicated that consumers satisfied with the tenderness of beef pay more attention to the taste of the meat. They found that flavor accounted for 49.4% of overall beef palatability, while tenderness and juiciness accounted for 43.4% and 7.4, respectively [13]. Similarly, Liu et al. [10] showed that taste (39%) and tenderness (31%), followed by juiciness (24%), were the most important attributes for European consumers evaluating beef samples from different countries.

The color of meat depends on its main heme pigment myoglobin. In addition to myoglobin, deoxymyoglobin, oxymyoglobin, metmyoglobin (MetMb) and sulfmioglobin contribute to the color change during meat cooking [48]. During cooking, the three forms of myoglobin undergo conversion and degradation through oxidation and reduction reactions, ultimately resulting in a dull brown color. However, the prevailing opinion is that the brown color of cooked meat is not necessary to prove that the meat has been sufficiently cooked [49]. Therefore, the color difference between cooked and sous vide meat can be observed even at temperatures differing by only 5 °C (between 60 and 65 °C; between 65 and 70 °C) [50].

Color and juiciness are, along with tenderness, the main sensory properties of beef. Juiciness is related to the variation of water content in meat during cooking, which also determines cooking efficiency, a critical factor for the industry [51]. The juiciness of meat in the present study was influenced by the type of heat treatment, and meat cooked sous vide had more favorable ratings (Table 5).

The right balance of juiciness and tenderness is important in meeting consumer satisfaction and expectations of the product. Further research into consumer sensory perception and acceptance of low-quality beef (especially from older animals) cooked under sous vide conditions would be of great importance in determining the utility and consumer acceptance of this technique [15].

## 4. Materials and Methods

The experimental material consisted of samples of the longest thoracic muscle (musculus longissimus thoracis, MLT) and supraspinatus muscle (musculus semitendinosus, MS) obtained from beef half-carcasses. The muscles were obtained from 18 carcasses of heifers derived from crossing Polish Holstein-Friesian cows with Limousine bulls. The average age of the animals ranged from 19–22 months and the live weight was 535.4 ± 66.5 kg. The animals came from one farm and were kept in a semi-intensive system. During the summer season, the main feed was green fodder and corn silage, while during the winter season corn silage predominated. In addition, the animals received meadow hay and ground grain. Information on breed and housing conditions was obtained from slaughterhouse purchase documents. Slaughtering was carried out in accordance with meat industry practice. The carcasses were subjected to electrostimulation, and after the assessment according to the EUROP system (European Union beef carcass classification system), they were qualified in terms of forming as the following classes: O (50%) and R-(50%), while in terms of fatness as class 3 (100%). The obtained muscle samples were vacuum packed and transported (+3 ± 0.5 °C) to the laboratory. Tests were conducted after 2 and 21 days of cold storage (+3 ± 0.5 °C). The meat was evaluated raw and cooked traditionally and heat-treated by the sous vide method.

### 4.1. Heating and Storage

The obtained material was divided into samples weighing approximately 322 ± 40 g. The methodology was developed based on the work of Baldwin [21], in which the adopted heat treatment conditions allow the obtaining of a redder color of cooked meat. The weighed samples were packed in sous vide bags and heat-treated in water at 65 °C for 2 h. Samples after the heat treatment were cooled to 4 °C and tested. The second part of the samples was subjected to conventional cooking at 96 °C until reaching an internal temperature of 65 °C. The weight ratio of water to meat was 2:1.

### 4.2. Meat Color

Meat color was determined based on the values of the parameters L*, a*, b* in the CIE LAB system [52] using the reflection method with an NR20XE camera (3nh Technology Co. Ltd. Shenzen, China); light source D65, measuring head aperture 20 mm, calibration with white standard: L*—99.18, a*—0.07, b*—0.05. In this system, L* stands for brightness, which is a spatial vector, while a* and b* are trichromaticity coordinates, where positive values of a* correspond to red and negative values to green, and positive values of b* correspond to yellow and negative values to blue. Measurements were taken at three locations of each sample of raw meat, as well as meat after traditional cooking and sous vide after cooling on a cross-section of the sample after half-hour storage at 4 °C.

The absolute color difference between the tested muscles was calculated using the formula:

$$\Delta E=\sqrt{{\left(\Delta L\right)}^{2}+{\left(\Delta a\right)}^{2}+{\left(\Delta b\right)}^{2}\;}$$

It can be assumed that the observer notices the color difference as follows:

- 0 < Δ*E* < 1—does not notice the difference,
- 1 < Δ*E* < 2—only an experienced observer notices the difference,
- 2 < Δ*E* < 3.5—an inexperienced observer also notices the difference,
- 3.5 < Δ*E* < 5—notices a distinct color difference,
- 5 < Δ*E*—an observer gets the impression of two different colors

The shear force was measured using a TA.XT plus texturometer (Stable Micro Systems Ltd., Surrey, UK) equipped with a Warner–Bratzler shear blade with a triangular cut. The samples of raw meat were cut using a cylinder-shaped cork borer (with a diameter of 12.7 mm) along the muscle fibers. The samples prepared in this way were cut into sections, and the shear force (N/cm^2^) applied during the cutting process was recorded. Three technical repetitions were carried out per sample.

### 4.3. Instrumental Texture Evaluation

Three 20 mm × 20 mm × 20 mm cubes were extracted from each sample. Instrumentally, the texture parameters of the meat samples were determined using Texture Profile Analysis (TPA) performed with a Texture Analyzer-CT3-25 (Brookfield, Wisconsin, USA), with a 38.1 mm diameter, 20 mm long cylindrical attachment. A two-fold compression test was performed on the samples to 50% of their height. The speed of the cylinder during the test was 2 mm/s, while the interval between pressures was 2 s. The following parameters were measured using Texture Pro CT software (Brookfield, Wisconsin, USA): cycle 1 hardness, cycle 2 hardness, resilience, gumminess and chewiness. During serial measurements, all texture parameters were counted automatically.

### 4.4. Sensory Evaluation

Sensory evaluation of meat was carried out by a five-member team with proven sensory sensitivity in accordance with ISO 8586-2 [53] and ISO 8587 [54] in a laboratory organized in accordance with ISO 8589 [55]. A five-point grading scale extended by half notes was used to evaluate meat, taking into account the following quality distinguishing factors: aroma (intensity and desirability), juiciness, tenderness and palatability (intensity and desirability). For each distinguishing characteristic, each degree of the scale was assigned a corresponding definition of quality (1—bad, 2—insufficient, 3—sufficient, 4—good, 5—very good).

### 4.5. Statistical Analysis

Statistical analysis was conducted using the Statistica 13.3PL package from TIBCO Software Inc (Palo Alto, CA, USA). The effects of muscle type and storage time, as well as interactions between these factors, on the level of analyzed raw beef quality parameters were determined using a two-factor analysis of variance. In order to determine the differences in selected physicochemical properties of beef, which were influenced by the type of muscle (m. semitendinosus and m. longissimus thoracis), the thermal treatment used (traditional cooking and sous vide cooking) and cold storage time (2 days and 21 days), the data were analyzed using three-factor analysis of variance along with their interaction. To determine the effect of these parameters on the quality of the final product, the GLM (General Linear Model) procedure (ANOVA, STATISTICA v. 13.1; StatSoft, Krakow, Poland) was used for the fixed effect model. Significance of differences was determined using Tukey’s HSD test at a significance level of *p* < 0.05.

## 5. Conclusions

Based on the analysis of the results, it can be concluded that the quality of beef products is determined, among other factors, by the degree of maturity of the meat, the type of heat treatment used and the type of muscle. Beef meat subjected to sous vide heat treatment compared to traditional cooking was characterized by more favorable texture parameters: hardness 1, gumminess and cutting force. Cold storage of meat for 21 days contributed to the improvement of these characteristics in the tested muscles subjected to both traditional cooking and sous vide treatment. The cutting force, chewiness, or gumminess of sous vide-treated meat took more favorable values even after two days of storage compared to meat stored for 21 days and cooked traditionally. Meat after sous vide treatment showed significantly higher values of the color parameter a* indicating the proportion of red in the meat. The type of heat treatment, the type of muscle tested and the storage time were all factors influencing the noticeable difference in the colors of the meat evaluated. The use of low-temperature heat treatment for as little as 2 h yields positive results in the sensory evaluation of juiciness, tenderness, or palatability of beef meat.

## Figures and Tables

**Table 1:** Texture parameters and hydration properties of raw beef as a function of muscle type and cold storage time.

Specification	Unit of Measure	Muscle	Storage Time (Days)				ANOVA	*p*
			2		21			
			$\bar{x}$	se	$\bar{x}$	se		
Hardness Cycle 1	N	MS	151.71	8.41	140.80	9.18	-	-
		MLT	146.33	4.71	136.33	8.93		
Adhesiveness	mJ	MS	2.24 ^a^	0.38	3.63	0.35	S	0.003
		MLT	2.52	0.63	4.04 ^b^	0.49		
Resilience		MS	0.20 ^a^	0.02	0.22 ^ac^	0.02	M	0.0001
		MLT	0.14 ^ab^	0.01	0.12 ^b^	0.01		
Hardness Cycle 2	N	MS	37.51	13.71	76.27	14.51	-	-
		MLT	48.27	11.22	40.66	10.00		
Cohesiveness		MS	0.34 ^a^	0.03	0.41 ^a^	0.02	M	0.0001
		MLT	0.22 ^b^	0.02	0.22 ^b^	0.02		
Springiness	mm	MS	4.13 ^a^	0.43	4.94 ^ab^	0.23	M	0.0001
		MLT	3.00 ^ac^	0.27	2.83 ^b^	0.24		
Gumminess	N	MS	53.53	6.64	56.01	3.82	M	0.020
		MLT	31.99	3.46	43.37	11.59		
Chewiness	mJ	MS	262.09 ^a^	48.41	284.03 ^a^	27.19	M	0.0001
		MLT	105.42 ^b^	17.02	98.74 ^b^	11.79		
Shear force	N/cm^2^	MS	123.61 ^a^	5.78	157.94 ^b^	8.76	M S M × S	0.001 0.002 0.022
		MLT	88.98 ^c^	3.21	94.37 ^c^	5.75		
Cooking loss	[%]	MS	32.37 ^a^	0.11	24.50 ^b^	0.22	S	0.0001
		MLT	32.64 ^a^	0.1	24.42 ^b^	0.12		
Drip loss	cm^2^	MS	6.07	0.41	4.18	0.39	S	0.0001
		MLT	6.89	0.31	4.03	0.25		

ANOVA: two-factor analysis of variance between the muscle (M) and cold storage time (S); MLT: longissimus thoracis muscle; MS: semitendinosus muscle; ^a,b,c^—statistically significant differences at *p* < 0.05.

**Table 2:** Color parameters of raw beef depending on the type of muscle and cold storage time.

Specification	Muscle	Storage Time (Days)				ANOVA	*p*
		2		21			
		$\bar{x}$	se	$\bar{x}$	se		
L*	MS	41.19 ^a^	0.45	38.42 ^ab^	1.00	M	0.0001
	MLT	34.99 ^c^	0.89	35.36 ^bc^	1.14		
a*	MS	22.81 ^a^	0.71	23.50 ^a^	0.78	M S	0.009 0.012
	MLT	19.94 ^b^	0.44	22.74 ^a^	0.44		
b*	MS	9.74 ^a^	0.50	9.23 ^a^	0.73	M M × S	0.0001 0.009
	MLT	6.93 ^b^	0.29	8.79 ^a^	0.44		
ΔE (MS-MLT)		8.68 ± 0.85		5.89 ± 0.55		S	0.010
ΔE (2 D–21 D)	MS	5.97 ± 0.74				-	-
	MLT	7.14 ± 0.78					

ANOVA: two-factor analysis of variance between the muscle (M) and cold storage time (S); MLT: longissimus thoracis muscle; MS: semitendinosus muscle; ^a,b,c^—statistically significant differences at *p* < 0.05. D: days.

**Table 3:** Texture parameters of beef depending on the type of meat, cold storage time and thermal treatment used.

Specification		Muscle	GT				SV				ANOVA	*p*
			Storage Time (Days)									
			2		21		2		21			
			$\bar{x}$	se	$\bar{x}$	se	$\bar{x}$	se	$\bar{x}$	se		
Hardness Cycle 1	N	MS	120.86 ^a^	10.73	108.48 ^a^	7.47	92.67	9.47	102.76 ^a^	14.41	M × T × S	0.005
		MLT	90.65	10.86	108.56 ^a^	8.25	100.28	12.51	57.76 ^b^	5.93		
Adhesiveness	mJ	MS	0.16 ^a^	0.04	0.13 ^a^	0.04	0.62	0.18	0.70	0.2	-	-
		MLT	0.46	0.27	0.18 ^a^	0.07	0.92 ^b^	0.22	0.37	0.12		
Resilience		MS	0.21	0.01	0.21	0.01	0.23 ^a^	0.00	0.18 ^b^	0.01	M × T × S	0.009
		MLT	0.23 ^ac^	0.01	0.20	0.01	0.20 ^bc^	0.01	0.19 ^b^	0.01		
Hardness Cycle 2	N	MS	87.12	13.58	96.94 ^a^	6.63	80.10	8.49	90.70 ^a^	12.98	-	-
		MLT	82.68	11.19	92.53 ^a^	7.01	82.19	9.41	48.38 ^b^	4.89		
Cohesiveness		MS	0.55 ^a^	0.01	0.53 ^a^	0.01	0.56 ^a^	0.00	0.48 ^b^	0.01	T × S	0.001
		MLT	0.55 ^a^	0.02	0.53 ^ac^	0.01	0.53 ^ac^	0.01	0.47 ^b^	0.01		
Springiness	mm	MS	6.02 ^a^	0.13	6.26 ^a^	0.11	5.90 ^ab^	0.09	5.41 ^b^	0.11	T × S M × S M × C	0.004 0.043 0.031
		MLT	6.12 ^a^	0.24	5.80 ^ab^	0.15	5.41 ^b^	0.13	4.67 ^c^	0.11		
Gumminess	N	MS	66.90 ^a^	6.64	57.69 ^a^	4.15	52.46 ^a^	5.47	50.56	7.94	M × T × S	0.018
		MLT	50.23	6.33	57.27 ^a^	4.32	50.96	5.20	27.49 ^b^	3.20		
Chewiness	mJ	MS	404.36 ^a^	41.78	359.10 ^a^	25.06	309.43 ^a^	33.50	273.65 ^a^	44.22	-	-
		MLT	313.09 ^a^	42.64	336.98 ^a^	28.44	270.51 ^a^	24.54	126.49 ^b^	15.22		
Shear force	N/cm^2^	MS	134.09 ^a^	3.37	116.02 ^b^	4.09	108.43 ^b^	4.07	99.43 ^bc^	2.38	T × S	0.049
		MLT	101.78 ^c^	2.97	87.06 ^cd^	3.45	74.87 ^cd^	2.95	69.77 ^cd^	3.05		

ANOVA: three-factor analysis of variance between the muscle (M), cold storage time (S) and type of heat treatment (T); MLT: longissimus thoracis muscle; MS: semitendinosus muscle; ^a,b,c,d^—statistically significant differences at *p* < 0.05; GT: traditionally cooked meat; SV: sous vide treated meat.

**Table 4:** Beef color parameters depending on the type of meat, cold storage time and thermal treatment used.

Specification	Muscle		GT				SV				ANOVA	*p*
			Storage Time (Days)									
			2		21		2		21			
			$\bar{x}$	se	$\bar{x}$	se	$\bar{x}$	se	$\bar{x}$	se		
L*	MS		53.24 ^a^	0.91	57.74 ^b^	0.42	58.49 ^bc^	1.16	62.08 ^c^	0.73	M × S	0.008
	MLT		50.98 ^a^	0.88	52.10 ^a^	0.84	56.51 ^ab^	1.1	56.68 ^ab^	0.9		
a*	MS		9.56 ^a^	0.16	10.03 ^a^	0.27	19.93 ^b^	0.54	15.91 ^c^	0.51	M × T × S T × S	0.026 0.0001
	MLT		10.67 ^a^	0.32	10.84 ^a^	0.32	20.22 ^b^	0.58	18.78 ^bd^	0.67		
b*	MS		12.21 ^a^	0.14	11.61 ^ac^	0.16	13.09 ^b^	0.22	11.36 ^c^	0.14	M × S T × S	0.046 0.0001
	MLT		12.14 ^ac^	0.16	12.09 ^ac^	0.13	13.06 ^b^	0.26	11.83 ^a^	0.22		
ΔE (2 D–21 D)	MS		4.80 ± 0.67 ^a^				6.66 ± 0.68				M T	0.024 0.026
	MLT		3.61 ± 0.42				4.78 ± 0.83 ^b^					
ΔE (GT-SV)	MS	2 D	12.9 ± 0.46 ^a^								S S × M	0.0001 0.010
		21 D	7.76 ± 0.44 ^b^									
	MLT	2 D	11.8 ± 0.61 ^ab^									
		21 D	9.83 ± 0.82 ^b^									
ΔE (MS-MLT)	2 D		6.04 ± 0.77				8.16 ± 1.05				-	-
	21 D		6.57 ± 0.87				7.54 ± 1.14					

ANOVA: three-factor analysis of variance between the muscle (M), cold storage time (S) and type of heat treatment (T); MLT: longissimus thoracis muscle; MS: semitendinosus muscle; ^a,b,c,d^—statistically significant differences at *p* < 0.05; GT: traditionally cooked meat; SV: sous vide treated meat. D: days.

**Table 5:** Sensory properties of beef depending on the type of meat, cold storage time and thermal treatment used (points).

Specification	Muscle	GT				SV			
		Storage Time (Days)							
		2		21		2		21	
		$\bar{x}$	se	$\bar{x}$	se	$\bar{x}$	se	$\bar{x}$	se
Aroma-intensity	MS	2.94	0.11	3.19	0.12	3.42	0.16	3.36	0.11
	MLT	2.94	0.17	3.28	0.15	3.31	0.16	3.44	0.11
Aroma-desirability	MS	3.11	0.14	3.25	0.09	3.53	0.14	3.58	0.12
	MLT	3.08	0.19	3.36	0.13	3.39	0.15	3.44	0.18
Juiciness	MS	2.86 ^a^	0.13	3.00	0.19	3.47	0.14	3.50	0.16
	MLT	3.08	0.19	3.17	0.16	3.39	0.14	3.56 ^b^	0.17
Tenderness	MS	3.06 ^a^	0.17	3.47 ^abc^	0.13	3.53 ^abc^	0.16	3.92 ^b^	0.18
	MLT	2.61 ^a^	0.24	3.47 ^ab^	0.19	3.08 ^a^	0.17	3.72 ^b^	0.21
Tastiness-intensity	MS	3.39	0.13	3.36	0.15	3.69 ^a^	0.10	3.94 ^a^	0.14
	MLT	2.94 ^b^	0.19	3.44	0.21	3.36	0.14	3.92 ^a^	0.11
Tastiness-desirability	MS	3.47	0.15	3.42	0.16	3.69	0.10	3.86	0.15
	MLT	3.14	0.20	3.44	0.22	3.31	0.13	3.64	0.22

ANOVA: three-factor analysis of variance between the muscle (M), cold storage time (S) and type of heat treatment (T); MLT: longissimus thoracis muscle; MS: semitendinosus muscle; ^a,b,c^—statistically significant differences at *p* < 0.05; GT: traditionally cooked meat; SV: sous vide treated meat.

## Data Availability

The authors declare that data or models are not deposited in an official repository.

## Sample Availability

Samples of the compounds are not available from the authors.
